# Radiofrequency Ablation for Refractory Palmar and Plantar Hyperhidrosis: A Case Report

**DOI:** 10.5812/aapm-172975

**Published:** 2026-02-28

**Authors:** Karim Hemati, Shima Movassaghi, Parnian Hemati

**Affiliations:** 1 Department of Anesthesiology and Pain Medicine, Pian Research Center, Iran University of Medical Sciences, Tehran, Iran; 2 Peoples’ Friendship University of Russia (RUDN University), Moscow, Russia

**Keywords:** Primary Hyperhidrosis, Palmar Hyperhidrosis, Plantar Hyperhidrosis, Radiofrequency Ablation, Sympathetic Chain, Thoracic Sympathetic Chain, Lumbar Sympathetic Chain

## Abstract

**Introduction:**

Palmar and plantar hyperhidrosis (PH) is characterized by sweat production that far exceeds thermoregulatory needs and often has substantial effects on daily functioning, social interaction, and mental health. The condition results from sympathetically mediated overstimulation of the eccrine glands, which are innervated by unmyelinated cholinergic C-type fibers. Radiofrequency ablation (RFA) offers a minimally invasive approach to symptom control in refractory PH through heat-mediated disruption of these sympathetic fibers. Reports describing staged upper- and lower-limb RFA for combined palmar and plantar disease in a single patient remain uncommon; this is the primary reason for presenting this case.

**Case Presentation:**

A 21-year-old man (American Society of Anesthesiologists physical status I) presented with a 10-year history of progressive palmar and plantar hyperhidrosis that had not shown durable responses to conservative or prior interventional treatment. He used topical aluminum chloride for 2 months without meaningful benefit and subsequently underwent 2 sessions of palmar botulinum toxin injections 5 months apart, which reduced palmar sweating by an estimated 50 - 60% for approximately 2 months per session; the lower limbs were not treated. Eighteen months before the current admission, he underwent RFA of the right thoracic sympathetic chain at another center. This produced approximately 80% improvement in palmar sweating, although sweating persisted along the ulnar nerve distribution; the benefit lasted only approximately 4 months, after which symptoms recurred to baseline. During the present admission, staged RFA was performed bilaterally at L3, with different lower-limb protocols selected in advance based on the prior treatment response and the greater severity of plantar sweating on the left. Seven days later, bilateral RFA was performed at T3. At the 2-week, 1-month, and 7-month follow-up visits, the patient reported no compensatory sweating or change in cutaneous sensation. By his own assessment, overall sweating of the palms and soles was reduced by approximately 90% from baseline, with a satisfaction score of 9/10 and subjectively improved quality of life.

**Conclusions:**

Staged RFA targeting both the thoracic and lumbar sympathetic chains appears to be a reasonably safe palliative option for patients with refractory combined palmar and plantar hyperhidrosis, with a more favorable complication profile than surgical sympathectomy. This case is considered reportable because it involves combined, staged treatment of both the upper and lower limbs in a single patient.

## 1. Introduction

Primary palmar hyperhidrosis (PH) is a chronic condition in which the palms produce substantially more sweat than is required for physiological needs, often interfering with work, social life, and psychological well-being (1). It results from pathological overactivity of the sympathetic nervous system, leading to persistent excessive stimulation of the eccrine glands. Onset usually occurs in childhood or adolescence, and symptoms frequently persist into adulthood, causing sustained embarrassment and functional impairment (2). The eccrine glands are innervated by unmyelinated C-type sympathetic fibers, with acetylcholine as the principal neurotransmitter at this synapse (3).

First-line treatment is generally conservative. Intradermal botulinum toxin injection is the most commonly used nonsurgical option, along with topical aluminum chloride or glutaraldehyde solutions, iontophoresis, and pharmacological approaches such as oxybutynin, propranolol, antidepressants, propantheline bromide, biofeedback, and psychotherapy (4). Radiotherapy was used historically but was abandoned because of unacceptable dermatological toxicity (4).

In practice, however, topical agents, iontophoresis, and botulinum toxin tend to provide only temporary relief; therefore, many patients ultimately require a more definitive procedure for sustained control (2).

Among surgical options, video-assisted thoracoscopic sympathectomy (VATS) remains the dominant approach for PH (5). However, it requires general anesthesia, entails greater operative trauma than less invasive alternatives, and is associated with a substantial rate of postoperative compensatory hyperhidrosis (6), all of which complicate patient selection.

Radiofrequency ablation (RFA) offers a less invasive alternative, providing localized thermal coagulation with minimal injury to surrounding tissue and allowing a rapid return to normal activity (7, 8). It is generally associated with less postoperative pain and fewer severe complications than surgical sympathectomy; however, the long-term evidence base remains incomplete. Durability, recurrence, and patient-reported satisfaction have not been well characterized, and larger comparative studies are still needed (9).

## 2. Case Presentation

### 2.1. Ethical Considerations

Formal review by an institutional ethics committee was not required for this report.

Written informed consent for the case report and all associated images was obtained from the patient. He was informed that no identifying information would be disclosed and that all personal details would be removed from the manuscript.

### 2.2. Prior Conservative and Interventional Treatments

Before the current admission, the patient had undergone the treatments summarized in Table 1, in chronological order.

**Table 1.** Prior Conservative and Interventional Treatments Trialed Before the Current Admission

Treatments	Regimen / Duration	Anatomical Site Treated	Response (Patient-Reported)	Reason for Proceeding to Next Treatment
**Treatment 1: Topical aluminum chloride**	Daily topical application for 2 months	Palms and soles	No meaningful improvement; hyperhidrosis persisted, particularly in the soles	Inadequate response prompted a trial of botulinum toxin injection
**Treatment 2: Botulinum toxin injection**	2 sessions, 5 months apart	Palms only (lower limbs not treated)	Approximately 50 - 60% reduction in palmar sweating per patient report, lasting approximately 2 months after each session	Partial, short-lived effect and lack of a lower-limb treatment option prompted referral for RFA
**Treatment 3: RFA of the right thoracic sympathetic chain**	Single session at another facility, 18 months before the current admission	Right palm only (lower limbs not treated)	Approximately 80% reduction in palmar sweating per patient report, with residual sweating in the ulnar nerve distribution; benefit lasted only approximately 4 months, after which sweating recurred to pretreatment levels	Recurrence of palmar sweating and untreated plantar hyperhidrosis prompted referral for staged bilateral thoracic and lumbar RFA

The patient was a 21-year-old man with American Society of Anesthesiologists physical status I and a 10-year history of progressive bilateral hyperhidrosis affecting the hands and feet, refractory to the treatments summarized in Table 1. Approximately 18 months before this admission, he had undergone RFA of the right thoracic sympathetic chain at another facility. This produced an estimated 80% reduction in palmar sweating; however, sweating persisted along the ulnar nerve distribution. The improvement lasted only approximately 4 months, after which symptoms returned to pretreatment levels. The lower limbs were not addressed at that time.

### 2.3. Procedure

**Figure 1.**
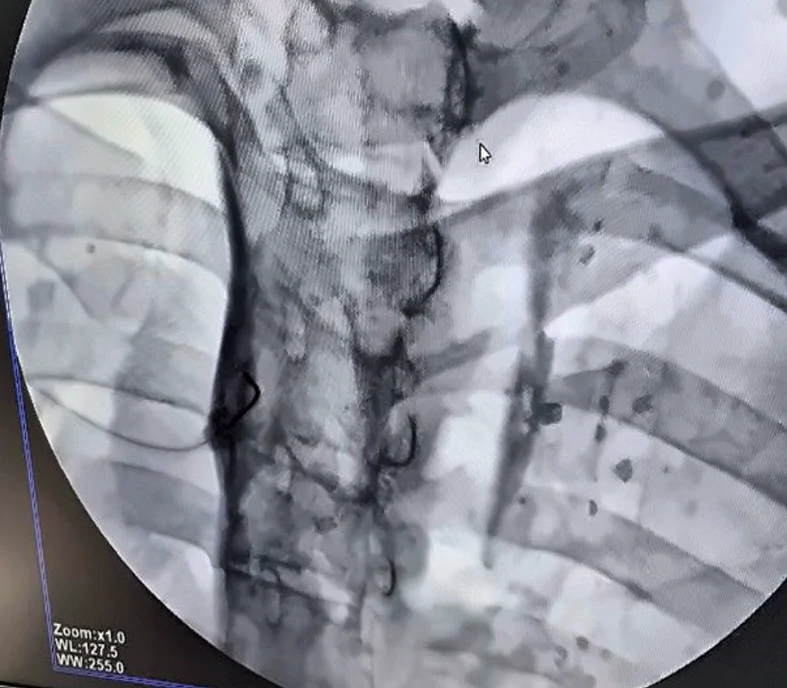
Lateral fluoroscopic view showing the radiofrequency needle in position at the L3 level, with contrast medium outlining the lumbar sympathetic chain

**Figure 2.**
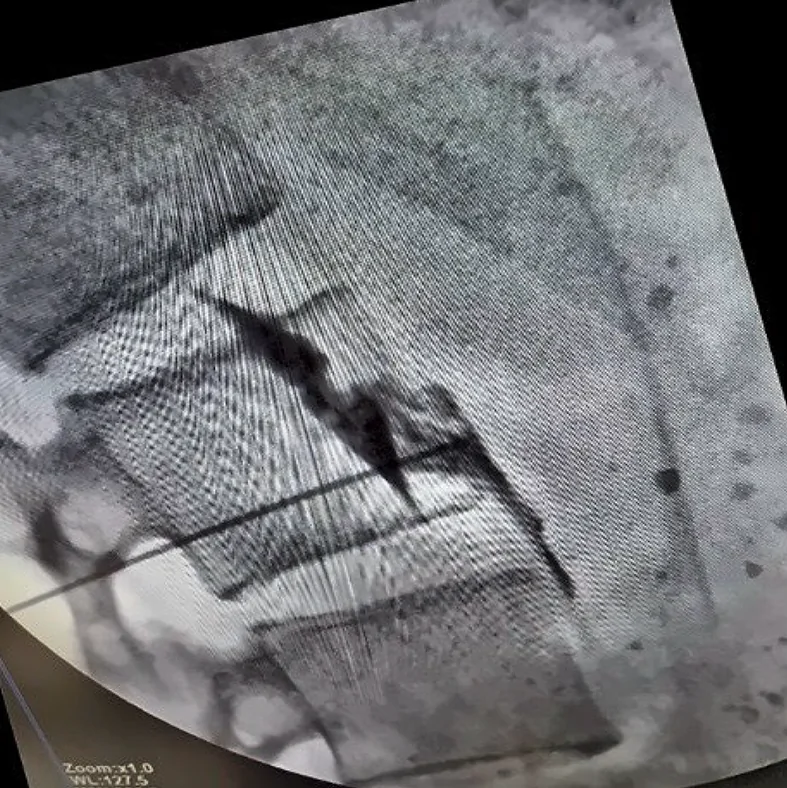
Lateral fluoroscopic view demonstrating needle placement and contrast medium distribution within the lumbar sympathetic chain

During the current admission, bilateral lower-limb RFA targeting the L3 lumbar sympathetic chain was performed in a single session under fluoroscopic guidance (Figures 1 and 2). Sedation consisted of intravenous midazolam 0.5 mg and fentanyl 50 μg. A 10-cm radiofrequency cannula with a 5-mm active tip was advanced to the L3 sympathetic chain using a Cosman radiofrequency generator (Cosman Medical, Burlington, MA, USA). Correct needle position was confirmed at each level by slowly injecting 1 mL of nonionic, water-soluble contrast medium (Visipaque 320) under live fluoroscopy and confirming spread along the sympathetic chain before each lesion. Tissue impedance during lesioning ranged from 200 to 400 Ω. One lesion was created at the lumbar level on each side.

Because the patient’s plantar sweating was subjectively worse on the left and given the partial, short-lived response to earlier thoracic RFA, two different lower-limb protocols were planned in advance rather than determined intraoperatively. The right lower limb received pulsed radiofrequency (RF) at 42°C for 5 minutes followed by conventional RF at 60°C for 1 minute, whereas the left, more severely affected side, received two cycles of conventional RF at 80°C for 90 seconds each, a more ablative regimen matched to its greater symptom burden.

Intraoperative skin-temperature monitoring of the lower limbs showed a decrease from a baseline of approximately 30°C to 27°C during lesioning, consistent with successful sympathetic interruption; the temperature continued to decrease to approximately 25°C during recovery-room monitoring. Approximately 30 minutes after the procedure, the patient reported a subjective reduction in lower-limb sweating.

**Figure 3.**
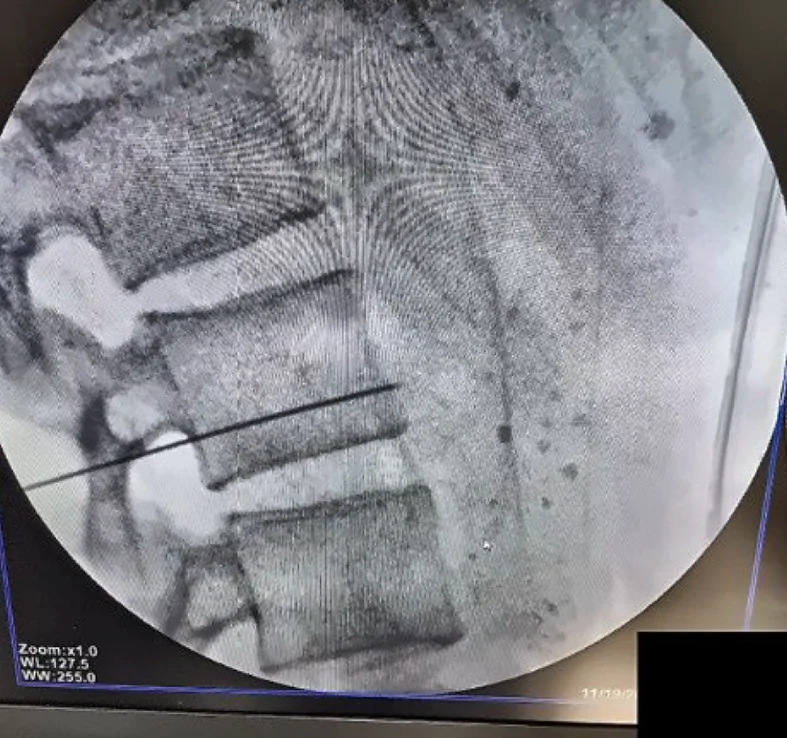
Anteroposterior fluoroscopic view demonstrating contrast medium spread along the thoracic sympathetic chain

Seven days later, bilateral upper-limb RFA was performed at the T3 level (Figure 3) using the same generator and needle specifications. Needle position was again confirmed by contrast spread along the thoracic sympathetic chain, and one conventional RF lesion was created on each side at 80°C for 90 seconds.

### 2.4. Outcome and Follow-Up

Outcome was assessed by patient self-report rather than by a validated severity instrument or objective measurement.

At the 2-week, 1-month, and 7-month follow-up visits, the patient reported no compensatory sweating and no change in cutaneous sensation. By his own assessment, overall sweating of the palms and soles was reduced by approximately 90% from baseline. He rated his satisfaction with this treatment, relative to earlier treatments, as 9 out of 10 and described his quality of life as subjectively improved. No procedure-related complications were noted at any point during follow-up.

## 3. Discussion

Hyperhidrosis results from excessive perspiration driven by an overactive sympathetic ganglion supplying the affected dermatome. RFA and surgical sympathectomy remain the two principal interventional options; however, robust head-to-head data on their comparative efficacy, safety, and durability remain limited (10).

The available comparative studies indicate a fairly consistent trade-off between efficacy and safety. In a retrospective cohort of 91 patients, sympathectomy produced a greater reduction in Hyperhidrosis Disease Severity Scale scores than RFA at both 6 and 12 months, with a longer overall duration of relief (11.8 vs 9.2 months); however, compensatory sweating was more common after sympathectomy (34.0% vs 18.2%), whereas recurrence was more common after RFA (27.3% vs 10.6%) (11). An earlier randomized comparison found a similar pattern: RFA achieved a 75% success rate compared with 95% for sympathectomy, with recurrence in 25% of RFA patients compared with 5% after surgery (12). Recurrence following RF sympathectomy also appears to increase over time. A 2023 comparison of computed tomography-guided radiofrequency with chemical sympathetic block reported that recurrence increased from approximately 6% at 1 month to more than two-thirds of patients by 3 years (13), underscoring that the 7-month follow-up reported here is too short to assess long-term durability.

Zhu et al. similarly found that sympathectomy provides durable relief but at the cost of a meaningfully higher rate of compensatory hyperhidrosis (14), and Han et al. reported compensatory sweating in up to 60% of sympathectomy patients (15). These findings help explain why many clinicians remain cautious about the surgical approach.

In a systematic review of surgical sympathectomy, Furlan et al. grouped adverse events into perioperative surgical complications (dyspnea, acute chest syndrome, wound pain, pneumothorax, and pleural effusion) and later effects of sympathetic nerve excision (axillary, shoulder, or back pain; compensatory sweating; bradycardia; nasal congestion; neural injury; and intercostal neuralgia) (16).

A separate consideration specific to VATS is that general anesthesia itself disrupts thermoregulation and lowers the vasoconstriction threshold (17), and that intraoperative changes in palmar temperature have not reliably predicted eventual clinical cure (16).

Taken together, several studies suggest a more favorable safety profile for RFA than for sympathectomy, with a lower incidence of compensatory hyperhidrosis and generally milder complications overall (9), representing an appealing trade-off for patients who prioritize safety over maximal efficacy. Because satisfaction in this population depends as much on the burden of adverse effects as on the degree of symptom control, some patients may reasonably prefer pulsed RF despite its higher recurrence rate (5).

Evidence specific to lumbar RFA for plantar disease, the technique used on this patient's feet, is considerably sparser than that for thoracic RFA in palmar hyperhidrosis. Early case reports described percutaneous lumbar sympathetic RF neurolysis for plantar sweating, with good short-term relief and minimal morbidity (18), and a comprehensive management review has since included RFA at both thoracic and lumbar levels among the minimally invasive options (4). However, controlled comparative data and longer-term recurrence rates specific to lumbar RFA remain scarce, which is an important limitation for the plantar component of this case. The procedural parameters used here, including tissue impedance of 200 - 400 Ω and a 5-mm active-tip electrode, are consistent with typical RFA technique and support the reproducibility of the approach. A recent case report combining pulsed and conventional radiofrequency in a single session for a different condition (Morton's neuroma) describes a similar dual-protocol logic, pairing pulsed RF for its lower-risk neuromodulatory effect with conventional RF for a greater lesion effect. This approach mirrors the mixed-protocol strategy used here, in which the more severely affected left foot received the more ablative conventional-only protocol (19).

### 3.1. Limitations

One limitation of this report, shared with much of the case-report literature on RFA for PH, is that improvement was judged by patient self-report rather than by a validated severity instrument or an objective measure such as gravimetric testing. We note this openly rather than presenting it as a formal outcome measure; future prospective series would benefit from standardized instruments such as the Hyperhidrosis Disease Severity Scale.

### 3.2. Conclusions

Overall, the literature suggests that RFA can serve as a reasonably safe palliative strategy for patients whose PH does not respond adequately to conservative therapy. This case adds to that literature by showing that staged bilateral RFA, combining thoracic and lumbar sympathetic targets in the same patient with follow-up to 7 months, can produce substantial improvement in both palmar and plantar sweating without observed complications. This combination of thoracic and lumbar, palmar and plantar treatment in a single patient is the main reason this case is reported.

RFA appears to be a reasonably safe palliative option for patients with palmar and plantar hyperhidrosis that has not responded to conservative management. In the patient described here, a staged bilateral approach targeting the lumbar and thoracic sympathetic chains produced marked symptom relief that persisted to 7 months, without observed complications. Larger comparative studies using validated outcome measures and longer follow-up are still needed to clarify RFA's place relative to surgical sympathectomy, particularly for lumbar RFA in plantar disease.

## Data Availability

The data supporting the findings of this case report (clinical history, procedural details, and imaging) are included within the article. Additional de-identified data are available from the corresponding author upon reasonable request, in compliance with patient confidentiality requirements.
